# Evaluation of a dual-energy computed tomography parameter –radiomics combined model for solitary pulmonary nodules

**DOI:** 10.3389/fonc.2026.1778055

**Published:** 2026-07-22

**Authors:** Xiaojing Kan, Bin Nan, Ruidi Ni, Congyan Wang, Yukun Pan, Doudou Liu, Yinghui Ge

**Affiliations:** Department of Radiology, Key Laboratory of Cardiac Imaging Medicine in Henan Province, Central China Subcenter of National Center for Cardiovascular Diseases, Henan Cardiovascular Disease Center, Fuwai Central-China Cardiovascular Hospital, Central China Fuwai Hospital of Zhengzhou University, Zhengzhou, China

**Keywords:** benign-malignant differentiation, dual-energy CT, machine learning, pulmonary nodule, radiomics

## Abstract

**Objective:**

To construct a dual-energy computed tomography (DECT) parameter–radiomics combined model based on DECT quantitative parameters and radiomic features and to evaluate its diagnostic performance in differentiating benign from malignant solitary pulmonary nodules.

**Materials and methods:**

We retrospectively collected data from inpatients who underwent contrast-enhanced thoracic DECT scans at our hospital between June 2018 and November 2024. Quantitative parameters measured and calculated included iodine concentration, effective atomic number, normalized iodine concentration, normalized effective atomic number, spectral Hounsfield unit slope, and extracellular volume fraction. These DECT parameters were used to construct a predictive model. The dataset was randomly divided into training and test sets in a 7:3 ratio. Radiomic features were extracted from mixed-energy images of the arterial and venous phases (A_120kVp_ and V_120kVp_) and from 70-keV monoenergetic images (A_70keV_ and V_70keV_). Six machine-learning algorithms were used to construct radiomics models, and the optimal model was selected based on performance. This optimal radiomics model was then combined with the DECT parameter model to create the combined model. Model performance was evaluated using accuracy, sensitivity, specificity, and area under the curve (AUC), and model comparisons were performed using the DeLong test (Python). A *P*-value of<0.05 was considered statistically significant.

**Results:**

In total, 209 patients were enrolled (166 in the malignant group and 43 in the benign group). In the test set, the diagnostic performance of the combined model (AUC = 0.906) exceeded that of the DECT-only radiomics model (AUC = 0.794). The combined model demonstrated an accuracy of 82.5%, sensitivity of 80.0%, and specificity of 92.3%.

**Conclusion:**

The DECT parameter–radiomics combined model effectively differentiated benign from malignant solitary pulmonary nodules and shows promising potential for clinical translation.

## 1 Introduction

Lung cancer remains the most common cancer and the leading cause of cancer-related mortality worldwide. Globally, approximately 2.48 million new lung cancer cases were reported, accounting for about 12.4% of all new cancer diagnoses, along with 1.8 million deaths (1). The age-standardized mortality rate for lung cancer is 16.8 per 100,000 population (1), notably higher than that of many other major malignancies. The overall 5-year survival rate is approximately 20%, largely due to delayed diagnosis and limited treatment responsiveness (2, 3). Conversely, patients diagnosed at an early stage (e.g., stage I or IA) have a 5-year survival rate exceeding 75%, whereas those with advanced disease (stage IV) have a survival rate below 5% (4). These data underscore the critical importance of early detection in improving outcomes and survival for patients with lung cancer.

The integration of low-dose computed tomography (CT) with artificial intelligence technology has markedly improved the sensitivity of pulmonary nodule detection. However, a high false-positive rate remains a major limitation of current screening systems. Therefore, developing more accurate noninvasive diagnostic methods has become an urgent priority in the early detection and diagnosis of lung cancer. Dual-energy CT (DECT) combined with radiomics can leverage the multiple quantitative parameters generated by DECT and extract high-throughput imaging features, including morphological, intensity, and texture information (5, 6). This combined approach offers a promising technical pathway for achieving precise, noninvasive diagnosis of pulmonary nodules (7). Although DECT provides diverse imaging datasets through dual-energy scanning, the potential of multiparameter fusion for pulmonary nodule evaluation has not yet been fully realized. Further research is needed to determine which DECT quantitative parameters have the greatest diagnostic value for distinguishing benign from malignant pulmonary nodules at an early stage and which imaging and machine-learning techniques are most effective for processing these multiparametric data. Additionally, novel DECT-derived parameters such as extracellular volume fraction (ECV), which already has well-established clinical applications in myocardial disease assessment, have not been systematically investigated in the context of early pulmonary nodule diagnosis and warrant further exploration.

This study addresses these gaps by developing a predictive model based on DECT quantitative parameters and another based on DECT radiomic features. These models were subsequently integrated into a combined model, and their diagnostic performance in distinguishing benign from malignant pulmonary nodules was evaluated.

## 2 Materials and methods

### 2.1 Study participants

Clinical and imaging data were retrospectively collected from hospitalized surgical patients who underwent DECT contrast-enhanced scans at our institution between June 2018 and November 2024. Patients were included if they had complete clinical data: including age, gender, BMI and hematocrit (%). In order to better reflect real-world clinical scenarios and highlight the diagnostic value of the model for solitary pulmonary nodules, we limited the baseline clinical covariates to age, sex, and BMI. Smoking status was excluded from the initial feature set due to a high rate of missing or unquantified data in the historical medical records, which could potentially introduce statistical bias. Additionally, hematocrit content was incorporated solely because it is a necessary prerequisite for the calculation of the extracellular volume (ECV); a solitary solid or part-solid pulmonary nodule (solid component ≥50%) without calcification on CT, measuring 10–30 mm in diameter, with intranodular cavitation or necrosis ≤50%; and a definitive pathological diagnosis. Exclusion criteria were prior treatment or invasive biopsy before DECT scanning, significant pleural effusion adjacent to the nodule or concurrent obstructive atelectasis, and severe artifacts compromising pulmonary image quality.

### 2.2 Examination method

A dual-source CT scanner (SOMATOM Force, Siemens Healthineers, Germany) was used, with tube voltages of 90 kV for tube A and Sn150 kV for tube B, automatic tube current modulation (Care Dose 4D), collimation of 192 × 0.6 mm, rotation time of 0.25 seconds, and pitch of 0.55. For contrast-enhanced scanning, a nonionic iodinated contrast agent, iopromide (370 mg/mL; Bayer Healthcare LLC, Germany), was administered via the antecubital vein at a dose of 1 mL/kg body weight and an injection rate of 3.0 mL/s, followed by a 30-mL saline flush at the same rate. The bolus-tracking technique was used, with a region of interest (ROI) placed in the descending aorta and a trigger threshold of 150 Hounsfield units (HU). The arterial-phase scan was initiated after an 8-second delay, and the venous-phase scan was performed after a 40-second delay. All images were reconstructed with a slice thickness of 1 mm and matrix size of 512 × 512 and then transferred to the Siemens post-processing workstation (syngo.via VB60) for subsequent analysis.

### 2.3 DECT image analysis

For each solitary pulmonary nodule, quantitative dual-energy CT parameters were measured using a multi-slice ROI pooling method. First, the slice displaying the maximum axial cross-section of the lesion was selected. A circular or tumor-conforming ROI was manually delineated to encompass approximately two-thirds of the lesion area, carefully avoiding areas of gross cavitation, calcification, necrosis, and adjacent large blood vessels. The identical ROI positioning and size were replicated on the immediate superior and inferior adjacent slices. The average value derived from these three sequential cross-sections was utilized as the final measurement for downstream analysis.

The following quantitative parameters were measured or calculated in both the arterial phase (AP) and venous phase (VP): iodine concentration (A_IC_ and V_IC_), effective atomic number (Z_eff_: A_Zeff_ and V_Zeff_), normalized iodine concentration (A_NIC_ and V_NIC_), normalized effective atomic number (A_nZeff_ and V_nZeff_), spectral Hounsfield Unit curve slope (λHU: A_λHU_ and V_λHU_), and extracellular volume fraction (A_ECV_ and V_ECV_).The relevant derivative parameters were calculated according to the following standardized mathematical formulas:

$NIC=IC_{lesion}/\;IC_{aorta}$

$nZ_{eff}=Z_{eff,lesion}/\;Z_{eff,aorta}$


$$ECV\left(\%\right)=\left(1-Hct\right)\times IC_{lesion}/IC_{aorta}\times 100\%$$

$$HU=\left(HU_{40keV}-HU_{100keV}\right)/60$$

where IC_lesion_ and IC_aorta_ represent the iodine concentrations of the lesion and the thoracic aorta on the same slice, respectively; Z_eff, lesion_ and Z_eff, aorta_ denote the effective atomic numbers; Hct represents the patient’s hematocrit; and HU_40keV_ and HU_100keV_ represent the CT attenuation values (in Hounsfield Units) of the lesion at virtual monoenergetic imaging energies of 40 keV and 100 keV, respectively.All image segmentations and parameter extractions were performed independently by two radiologists (Reader 1 and Reader 2, with 6 and 5 years of experience in thoracic imaging, respectively). Both readers were completely blinded to the clinical and final histopathological outcomes during the entire measurement workflow.

### 2.4 DECT radiomics analysis

Previous studies (8, 9) have shown that virtual monoenergetic images in the 60–70 keV range provide superior contrast-to-noise ratio and tissue contrast for thoracic imaging. Therefore, the original arterial and venous-phase data were reconstructed into 120 kVp–equivalent mixed-energy images (A_120kVp_ and V_120kVp_) and 70-keV monoenergetic images (A_70keV_ and V_70keV_). The DICOM files were exported and converted to NIFTI format (.nii.gz) using MRIcroGL software, followed by preprocessing with Python’s *nibabel* library. Voxel sizes were uniformly resampled to 1 × 1 × 1 mm^3^ using trilinear interpolation.

Three-dimensional (3D) volumes of interest (VOIs) were semi-automatically segmented with manual adjustment on the A_70keV_ and V_70keV_ images using ITK-SNAP software (version 4.2). The segmentation protocol mandated (1): comprehensive encapsulation of the solid tumor components, and (2) strict exclusion of adjacent cystic changes, macroscopic necrosis, atelectasis, and surrounding bronchovascular structures. The finalized VOI masks were subsequently propagated to the co-registered A_120kVp_ and V_120kVp_ images. All segmentations were meticulously reviewed and refined by a senior thoracic radiologist (with >10 years of experience) to guarantee anatomical accuracy.

Notably, a dual-dimensional approach was strategically implemented between conventional DECT parameter measurement (2D three-slice averaging) and radiomics feature extraction (3D whole-lesion VOI). For conventional DECT metrics, localized 2D sampling minimizes partial volume effects from the adjacent healthy lung parenchyma and alleviates respiratory motion artifacts at the nodule boundaries, ensuring pure attenuation sampling. Conversely, full 3D VOI segmentation is imperative for radiomics to thoroughly capture high-dimensional spatial heterogeneity and microstructural textural patterns across the entire tumor volume, which would be lost in simplified 2D profiles.

To mitigate single-observer segmentation bias and guarantee feature reproducibility, an interobserver reliability analysis was performed prior to downstream feature selection. A second independent radiologist segmented a randomized subset of 30 nodules. The interobserver intraclass correlation coefficients (ICCs) were calculated, and only robust radiomic features demonstrating excellent stability (ICC> 0.75) were retained for subsequent model training, while unstable features were discarded.

High-throughput feature extraction was executed on the stable VOIs using the open-source PyRadiomics toolkit (version 3.0.1), yielding 107 baseline radiomic features (14 morphological, 18 first-order histogram, and 75 higher-order texture features) per phase. To address potential data anomalies, missing values and outliers were imputed using the median method, followed by Z-score normalization to standardize all feature scales to a mean of 0 and a standard deviation of 1. Feature dimension reduction was subsequently conducted via a sequential two-stage framework: first, redundant and highly collinear features were eliminated using Pearson correlation analysis with a retention threshold of |r| ≥ 0.9; second, the least absolute shrinkage and selection operator (LASSO) regression model was constructed using the scikit-learn package (version 1.3.0). The optimal regularization parameter λ was determined using 5-fold cross-validation, ultimately selecting features with non-zero coefficients to build the final radiomics signature.

### 2.5 Combined model

Construction of the DECT Parameter Model: A logistic regression (LR) model was constructed using clinical indicators, conventional CT parameters, and DECT parameters that showed statistically significant differences as independent variables, with pulmonary nodule malignancy as the dependent variable.

**Figure 1:**
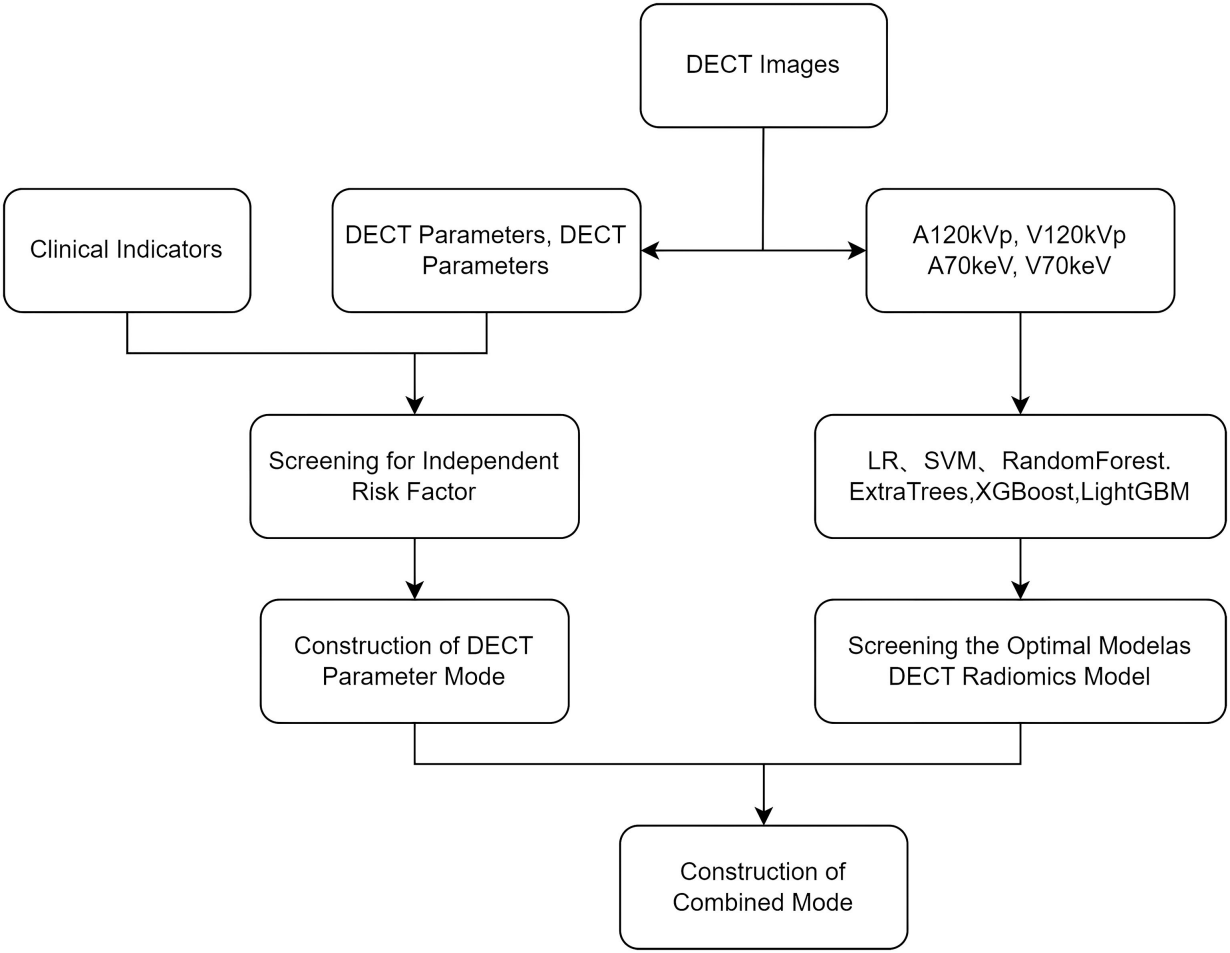
Flowchart of patient selection, inclusion/exclusion pathways, and dataset partitioning for the dual-energy CT radiomics model development. SPN, solitary pulmonary nodule.

Construction of the DECT Radiomics Model: A simple random sampling method (fixed random seed, seed = 20) was used to divide the samples into a training set and an independent test set in a 7:3 ratio. Based on the radiomics features extracted from the training set, six machine-learning algorithms were applied: LR, Support Vector Machine (SVM), Random Forest, ExtraTrees, XGBoost (eXtreme Gradient Boosting), and LightGBM (Light Gradient Boosting Machine). All modeling procedures were performed in Python 3.11. The LR, SVM, Random Forest, and ExtraTrees models were implemented using scikit-learn (version 1.3.0), while XGBoost and LightGBM were implemented using their respective dedicated Python libraries. Model training and validation were conducted using a 5-fold cross-validation strategy. First, the performance of models constructed with the six algorithms was compared across different image groups (A_120kVp_, V_120kVp_, A_70keV_, and V_70keV_). The best-performing model from each group was then compared to identify the optimal radiomics model. Finally, this optimal radiomics model was combined with the DECT parameter model to construct the DECT parameter–radiomics combined model (**Figure 1**).

### 2.6 Statistical analysis

Data were analyzed using SPSS (version 29.0) and R (version 4.3.1). Qualitative data are expressed as percentages (%) and were compared using the χ^2^ test or Fisher’s exact test. For quantitative data, normality was assessed using the Shapiro–Wilk test. Normally distributed quantitative variables are presented as mean ± standard deviation (x̄ ± SD), whereas non-normally distributed variables are reported as median and interquartile range [M (P25, P75)]. Interobserver agreement was evaluated using the ICC, with ICC< 0.50 considered poor, 0.50–0.75 moderate, 0.75–0.90 good, and >0.90 excellent. Group differences were assessed using the independent-samples *t*-test or the Mann–Whitney U test, with *P* < 0.05 indicating statistical significance. For the DECT parametric model, variable selection and model construction were performed using univariate and multivariate analyses. Model performance was evaluated based on accuracy, sensitivity, specificity, and area under the curve (AUC). Comparisons between models were performed using DeLong’s test (Python), with *P* < 0.05 considered statistically significant. A combined DECT parameter–radiomics model was developed using multivariate LR.

## 3 Results

### 3.1 Clinical data and CT parameters

Following screening under strict inclusion and exclusion criteria, 209 patients with solitary pulmonary nodules (SPNs) were enrolled (**Figure 1**), which were split into a training cohort (n = 146) for model building and a test cohort (n = 63) for model assessment. The cohort comprised 166 malignant and 43 benign SPN cases. The malignant group included 106 males (65.9%) and 60 females (34.1%), with mean age 65.0 ± 11.0 years and mean BMI 24.5 ± 3.69 kg/m²; the benign group contained 28 males (65.1%) and 15 females (34.9%), with mean age 61.60 ± 11.78 years and mean BMI 25.4 ± 3.43 kg/m². The mean age of the malignant group was significantly higher than that of the benign group (*P* < 0.05), whereas no significant differences were observed in sex, BMI, or hematocrit values (*P* > 0.05). Among the malignant cases, 135 were adenocarcinomas and 31 were squamous cell carcinomas. Concurrently, among the 43 benign cases, the histopathological subtypes and frequencies were distributed as follows: inflammatory granuloma (n = 19, 44.2%), organizing pneumonia (n = 15, 34.9%), tuberculous granuloma (n = 6, 14.0%), mucormycosis (n = 2, 4.6%), and hamartoma (n = 1, 2.3%). Regarding conventional CT parameters, the mean nodule diameter in the malignant group was significantly larger than that in the benign group (*P* < 0.05), whereas nodule location and nodule type showed no significant differences (*P* > 0.05) (**Tables 1A**, **1B**).

**Table 1A:** Baseline clinical characteristics of enrolled patients.

Characteristic	Benign (n=43)	Malignant (n=166)	*t*/*χ*^2^	P
Age (years)	59.6 ± 13.0	65.0 ± 11.0	-2.752	0.006*
Male	33(76.7%)	106(63.9%)	2.547	0.111
BMI(kg/m^2^)	25.4 ± 3.43	24.5 ± 3.69	1.340	0.182
Hematocrit (%)	39.98 ± 4.73	38.97 ± 5.01	1.200	0.232

*P<0.05 indicates a statistically significant difference.

**Table 1B:** Morphological and conventional CT characteristics of solitary pulmonary nodules.

Characteristic	Benign (n=43)	Malignant (n=166)	*t*/*χ*^2^	P
Nodule Location			4.298	0.636
Left Upper Lobe	8(18.6%)	41(24.7%)		
Left Lower Lobe	7(16.3%)	25(15.1%)		
Left Perihilar	0(0.00%)	9(5.42%)		
Right Upper Lobe	13(30.2%)	43(25.9%)		
Right Middle Lobe	3(6.98%)	12(7.23%)		
Right Lower Lobe	11(25.6%)	30(18.1%)		
Right Perihilar	1(2.33%)	6(3.61%)		
Nodule Type			0.086	0.769
Solid	39(90.7%)	148(89.2%)		
Subsolid	4(9.30%)	18(10.8%)		
Mean Diameter (cm)	1.92 ± 0.70	2.28 ± 0.68	-3.046	0.003*

*P<0.05 indicates a statistically significant difference.

### 3.2 DECT parameter model

**Figure 2:**
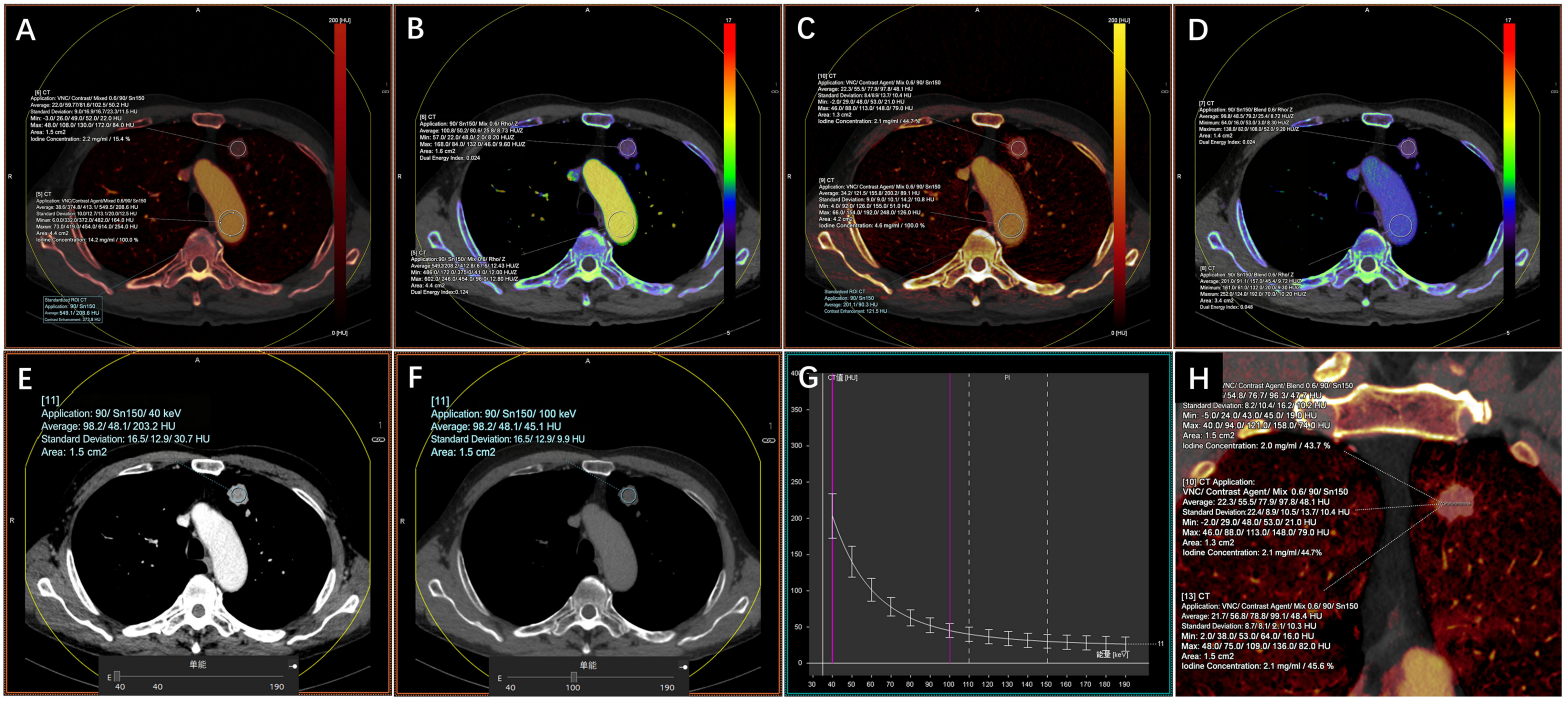
A 61-year-old male patient with lung adenocarcinoma presenting solitary pulmonary nodule. **(A–D)** show the arterial-phase iodine density map, arterial-phase effective atomic number (Zeff) map, venous-phase iodine density map, and venous-phase effective atomic number (Zeff) map reconstructed from DECT scans, respectively. **(E–G)** correspond to venous-phase 40 keV virtual monoenergetic image, venous-phase 100 keV virtual monoenergetic image, and spectral HU attenuation curve. **(H)** is the coronal reformatted image of the lesion, displaying quantitative measurements acquired on three consecutive slices; the average value of these three measurements was used for subsequent statistical analysis.

The multi-phase extraction workflow for conventional DECT quantitative parameters—including iodine density mapping, effective atomic number characterization, and spectral attenuation curve plotting—is schematically illustrated via a representative malignant adenocarcinoma case in **Figure 2**. As demonstrated, a localized three-slice averaging method (Panel H) was meticulously applied to minimize partial volume effects.

The interobserver ICC values for the DECT parameters ranged within 0.76–0.88 (95% confidence interval [CI]: 0.69–0.92) (**Supplementary Table 1**). Among these parameters, A_NIC_, A_ECV_, V_ECV_, and V_λHU_ showed significant differences between the benign and malignant groups (*P* < 0.05) (**Table 2**). Univariate analysis indicated that age (odds ratio [OR] = 1.040, 95% CI: 1.010–1.071, *P* = 0.008), mean diameter (OR = 2.081, 95% CI: 1.274–3.398, *P* = 0.003), A_NIC_ (OR = 0.002, 95% CI: 0.000–0.247, *P* = 0.012), A_ECV_ (OR = 0.864, 95% CI: 0.767–0.972, *P* = 0.015), V_ECV_ (OR = 0.764, 95% CI: 0.694–0.842, *P* < 0.001), and V_λHU_ (OR = 2.090, 95% CI: 1.234–3.539, *P* = 0.006) were significantly associated with pulmonary nodule malignancy. Multivariate analysis further demonstrated that mean diameter (OR = 2.513, 95% CI: 1.351–4.676, *P* = 0.004), V_ECV_ (OR = 0.733, 95% CI: 0.655–0.820, *P* < 0.001), and V_λHU_ (OR = 4.309, 95% CI: 2.236–8.303, *P* < 0.001) were independent predictors of pulmonary nodule malignancy.

**Table 2:** Comparison of DECT parameters of pulmonary nodules between the benign and malignant groups.

Characteristic	Benign(n=43)	Malignant(n=166)	*t*/*χ*^2^	P
Arterial phase				
IC(mg/ml)	1.62 ± 0.83	1.36 ± 0.76	1.934	0.054
NIC	0.14 ± 0.07	0.11 ± 0.06	2.358	0.022
nZ_eff_	0.72 ± 0.04	0.72 ± 0.45	-0.058	0.954
ECV(%)	5.30 ± 2.91	4.18 ± 2.48	2.549	0.012*
λHU	1.60 ± 0.66	1.78 ± 0.65	1.693	0.095
Venous phase				
IC(mg/ml)	1.78 ± 0.86	1.63 ± 0.67	1.041	0.302
NIC	0.40 ± 0.08	0.38 ± 0.12	1.503	0.136
nZ_eff_	0.89 ± 0.03	0.88 ± 0.04	1.715	0.088
ECV(%)	20.03 ± 3.14	14.72 ± 4.97	8.649	<0.001*
λHU	1.75 ± 0.75	2.11 ± 0.74	-2.816	0.005*

*P<0.05 indicates a statistically significant difference.

Based on the multivariate logistic regression analysis, the clinical-DECT combined model was constructed as follows:


$$Logit\left(P\right)=1.954+0.921\times Mean\;Diameter-0.311\times \left(V_{ECV}\right)+1.461\times \left(V_{\lambda HU}\right)$$

where P represents the predicted probability of malignancy. In this model, a larger mean diameter and a higher V_λHU_ functioned as independent risk factors for malignancy, whereas an elevated V_ECV_ was inversely associated with malignant risk. This quantitative formula provides an objective, noninvasive approach for characterizing solitary pulmonary nodules and serves as the baseline for subsequent hybrid model integration.

### 3.3 DECT radiomics model

**Figure 3:**
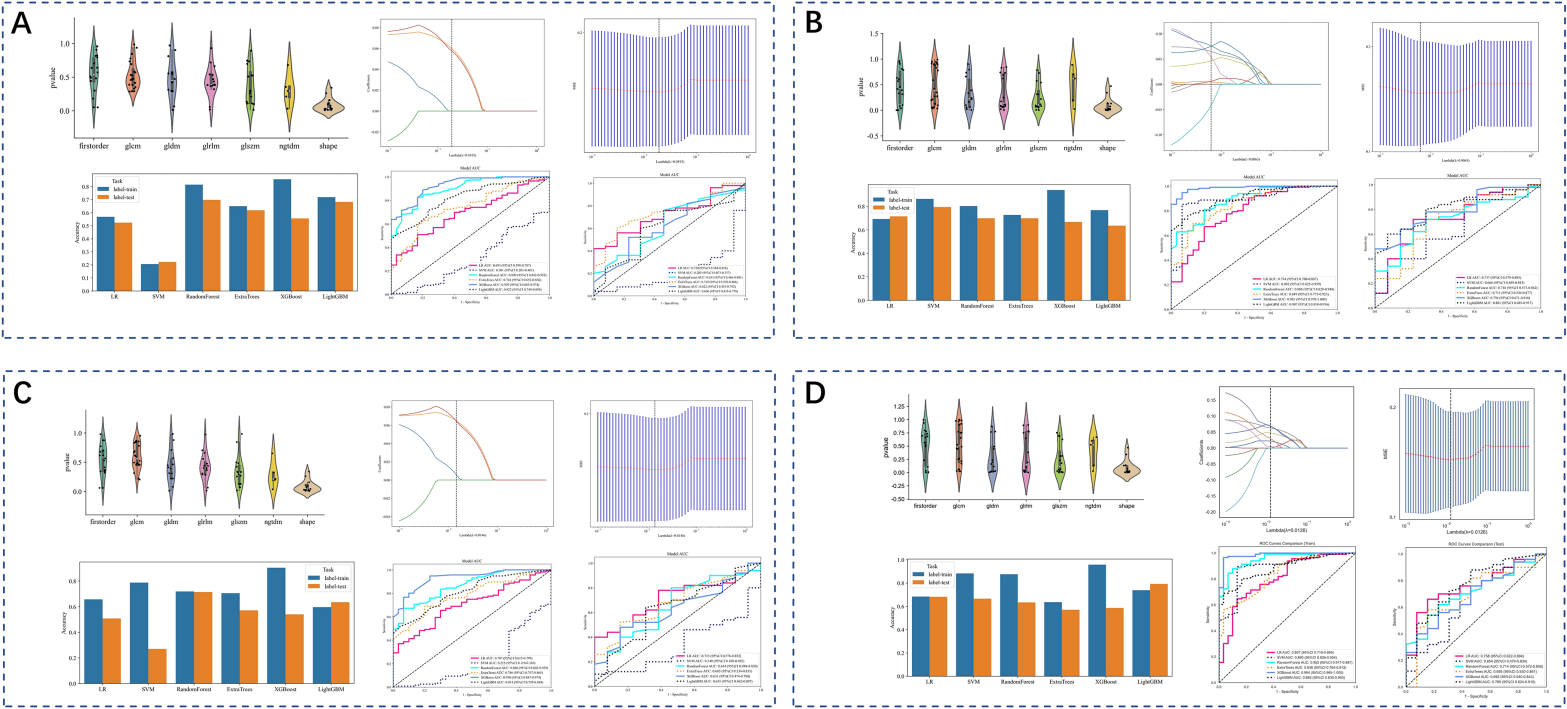
Radiomic feature screening and machine learning performance analyses for four types of DECT reconstructed images. **(A–D)** correspond to arterial-phase 70 keV virtual monoenergetic images, venous-phase 70 keV virtual monoenergetic images, arterial-phase mixed-energy images (equivalent to 120 kVp), and venous-phase mixed-energy images (equivalent to 120 kVp), respectively. Each panel contains six subgraphs: violin plots showing the distribution of extracted radiomic features; LASSO regularization coefficient profiles; curves illustrating the correlation between λ values and partial likelihood errors; bar charts comparing the accuracy of six machine learning classifiers (LR, SVM, RandomForest, ExtraTrees, XGBoost, LightGBM); ROC curves of the six models in the training cohort; and ROC curves of the six models in the validation cohort.

**Figure 4:**
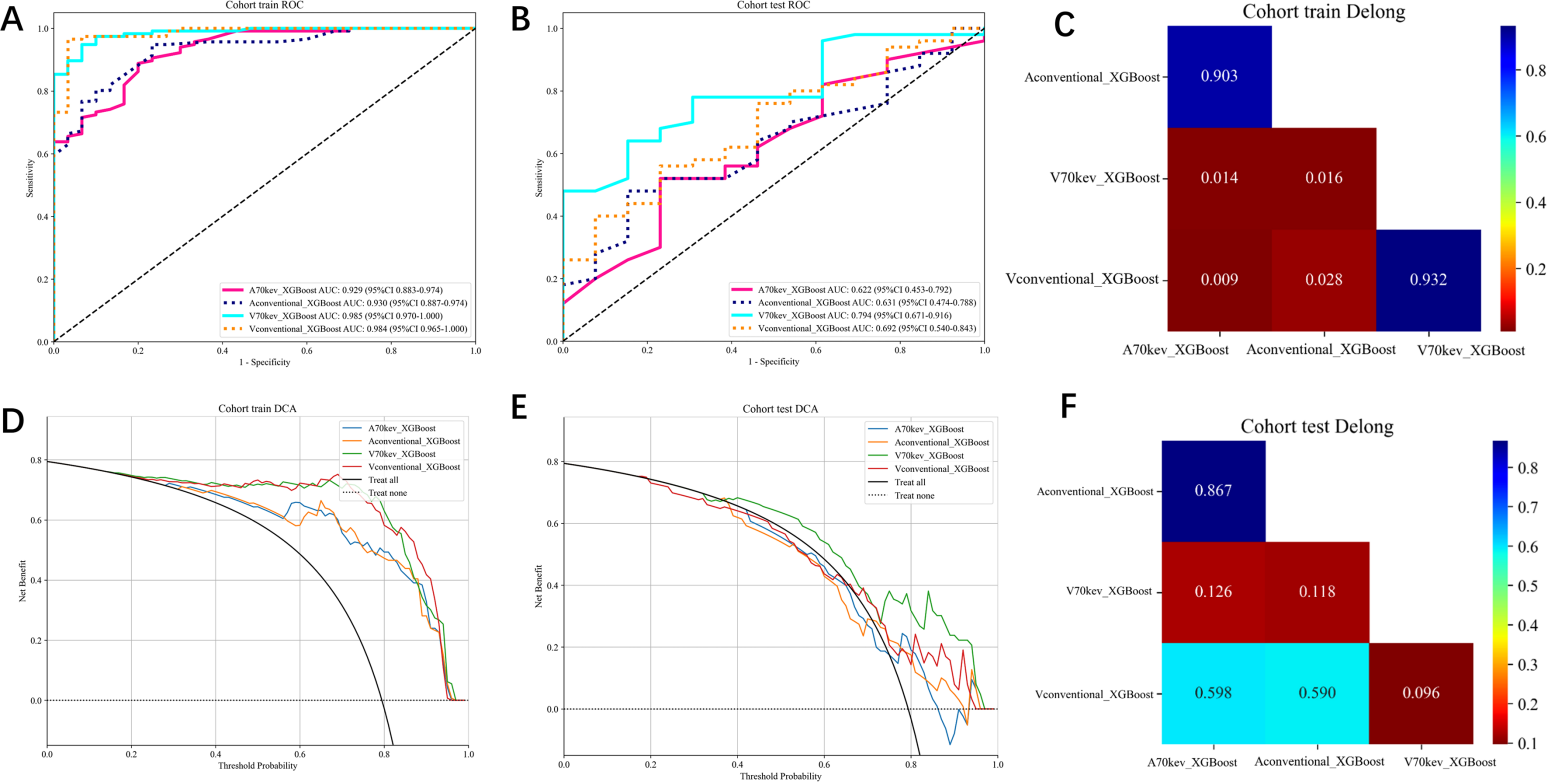
ROC curves, decision curve analysis (DCA), and DeLong test comparisons of radiomics models built on different DECT image types in the training and test cohorts. All models were constructed using the XGBoost algorithm. A70keV, Aconventional, V70keV, and Vconventional represent arterial-phase 70 keV virtual monoenergetic images, arterial-phase conventional mixed-energy images, venous-phase 70 keV virtual monoenergetic images, and venous-phase conventional mixed-energy images, respectively. **(A, B)** ROC curves of all models in the training cohort and test cohort, respectively. **(C, F)** Heatmap matrices of P values from DeLong test for the training cohort and test cohort; darker red corresponds to smaller P values (close to 0), while dark blue represents larger P values. **(D, E)** DCA curves of all models in the training cohort and test cohort, respectively.

High-throughput radiomic features derived from four DECT reconstruction series were robustly screened via LASSO regression, with violin plots of feature distributions, LASSO coefficient trajectories and optimal λ selection curves presented in separate panels of **Figure 3**. All enrolled samples were randomly split into a training cohort (146 lesions: 30 benign, 116 malignant) and a test cohort (63 lesions: 13 benign, 50 malignant) at a 7:3 allocation ratio. Preliminary comparison of six machine learning classifiers (LR, SVM, Random Forest, ExtraTrees, XGBoost, LightGBM) across the four image subgroups revealed that XGBoost yielded superior discrimination performance in both training and test sets (**Figure 3**). Further quantitative evaluation of XGBoost models built on A_70keV_, V_70keV_, A_120kVp_ and V_120kVp_ images in the test set identified the V70keV-based model as the optimal predictor, with an AUC of 0.794 (95% CI, 0.671–0.916), accuracy of 0.667, sensitivity of 0.620, and specificity of 0.846. The V_120kVp_ XGBoost model ranked second (AUC = 0.692; 95% CI, 0.540–0.843), accompanied by an accuracy of 0.587, sensitivity of 0.540, and specificity of 0.769 (**Table 3**). Pairwise AUC comparisons in the test set showed no statistically significant differences between the V_70keV_ model and the A_70keV_ (P = 0.128), A_120kVp_ (P = 0.118), or V_120kVp_ (P = 0.096) models. Likewise, no intergroup disparities were detected between the A_120kVp_ and V_120kVp_ models (P = 0.596), nor between the A_70keV_ and V_120kVp_ models (P = 0.500) (**Figure 4**).

**Table 3:** Diagnostic performance of the four DECT-based XGBoost radiomics models.

Dataset		Model	AUC	95% CI	Sensitivity	Specificity	Accuracy	Threshold
Training	A70kev_XGBoost		0.929	0.883~0.974	0.871	0.800	0.856	0.686
	Aconventional_XGBoost		0.930	0.887~0.974	0.940	0.767	0.904	0.650
	V70kev_XGBoost		0.985	0.970~1.000	0.940	0.933	0.938	0.735
	Vconventional_XGBoost		0.984	0.965~0.996	0.957	0.967	0.959	0.696
Test	A70kev_XGBoost		0.622	0.453~0.792	0.500	0.769	0.556	0.791
	Aconventional_XGBoost		0.631	0.474~0.788	0.460	0.846	0.540	0.696
	V70kev_XGBoost		0.794	0.671~0.916	0.620	0.846	0.667	0.784
	Vconventional_XGBoost		0.692	0.540~0.843	0.540	0.769	0.587	0.813

**Figure 5:**
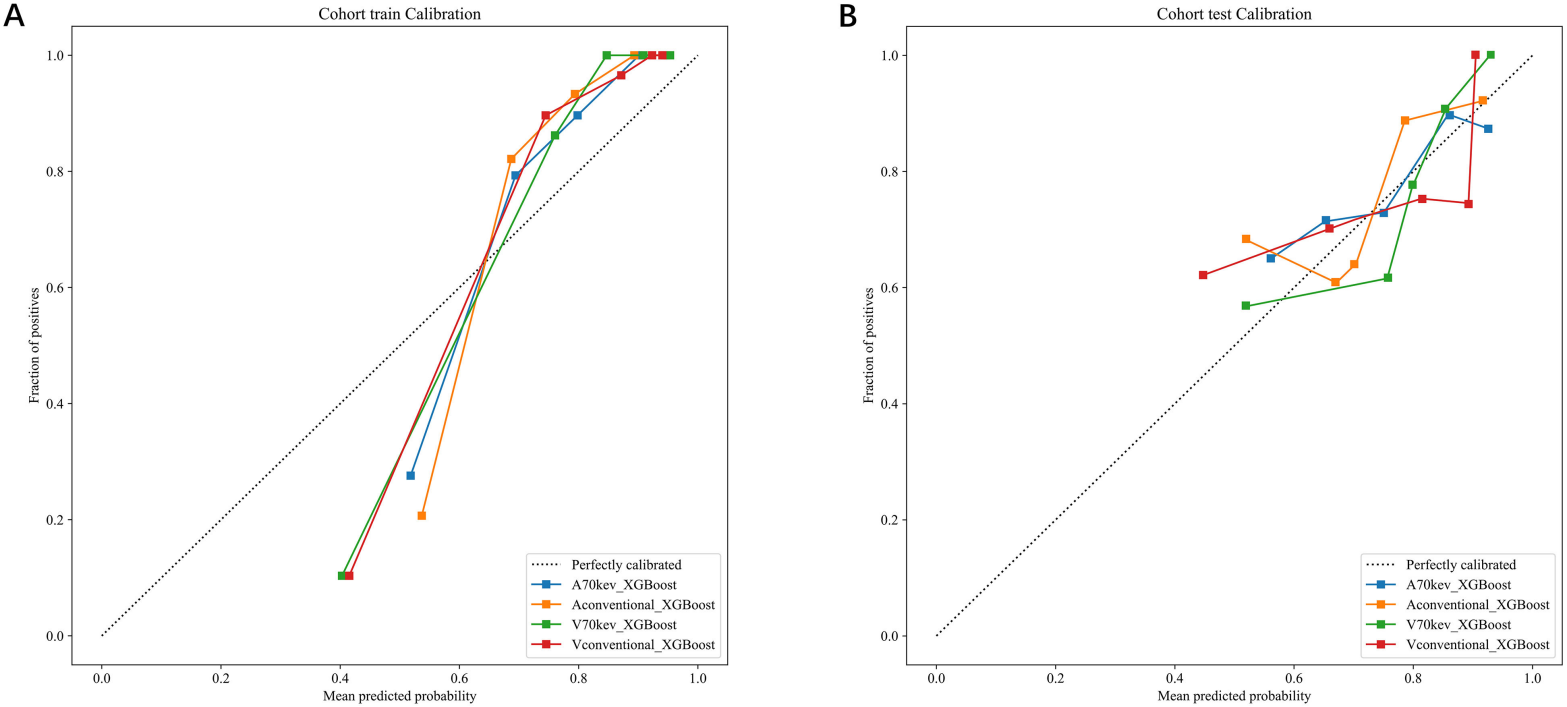
Calibration curves of dual-energy CT radiomics models in training and test cohorts. Calibration curve plots demonstrate the calibration between predicted risks of malignancy and observed outcomes of malignancy across four separate XGBoost classifiers (A70kev_XGBoost, Aconventional_XGBoost, V70kev_XGBoost, and Vconventional_XGBoost) in the training cohort **(A)** and the test cohort **(B)**. The black diagonal dotted line represents a perfect calibration where predicted probabilities exactly match observed frequencies.

The calibration of the four DECT-based radiomics signatures was assessed via calibration curve plots (**Figure 5**). The curve trajectories demonstrated exceptional agreements between the predicted risks of malignancy and the actual histopathological outcomes in both the training cohort (**Figure 5A**) and the independent test cohort (**Figure 5B**).

### 3.4 Combined model

A combined model was constructed by integrating the clinical features and DECT parameter model with the DECT radiomics model. In the training set, the combined model achieved an accuracy of 97.3% and AUC of 0.998 (95% CI: 0.995–1.000), with sensitivity and specificity of 0.966 and 1.000, respectively, at a threshold of 0.889. In the test set, the combined model demonstrated an accuracy of 0.825 and AUC of 0.906 (95% CI: 0.826–0.986), with sensitivity and specificity of 0.800 and 0.923, respectively, at a threshold of 0.987. These performance metrics were superior to those of the DECT radiomics model; however, the difference was not significant (*P* = 0.068) (**Table 4**).

**Table 4:** Diagnostic performance comparison between the DECT radiomics model and the combined model in the training and testing cohorts.

Dataset	Model	AUC	95% CI	Sensitivity	Specificity	Accuracy	Threshold
Training	DECT Radiomics Model	0.985	0.970~1.000	0.940	0.933	0.938	0.735
	Combined Model	0.998	0.995~1.000	0.966	1.000	0.973	0.889
Test	DECT Radiomics Model	0.794	0.671~0.916	0.620	0.846	0.667	0.784
	Combined Model	0.906	0.826~0.986	0.800	0.923	0.825	0.987

DECT, dual-energy computed tomography; AUC, area under the receiver operating characteristic curve; CI, confidence interval.

**Figure 6:**
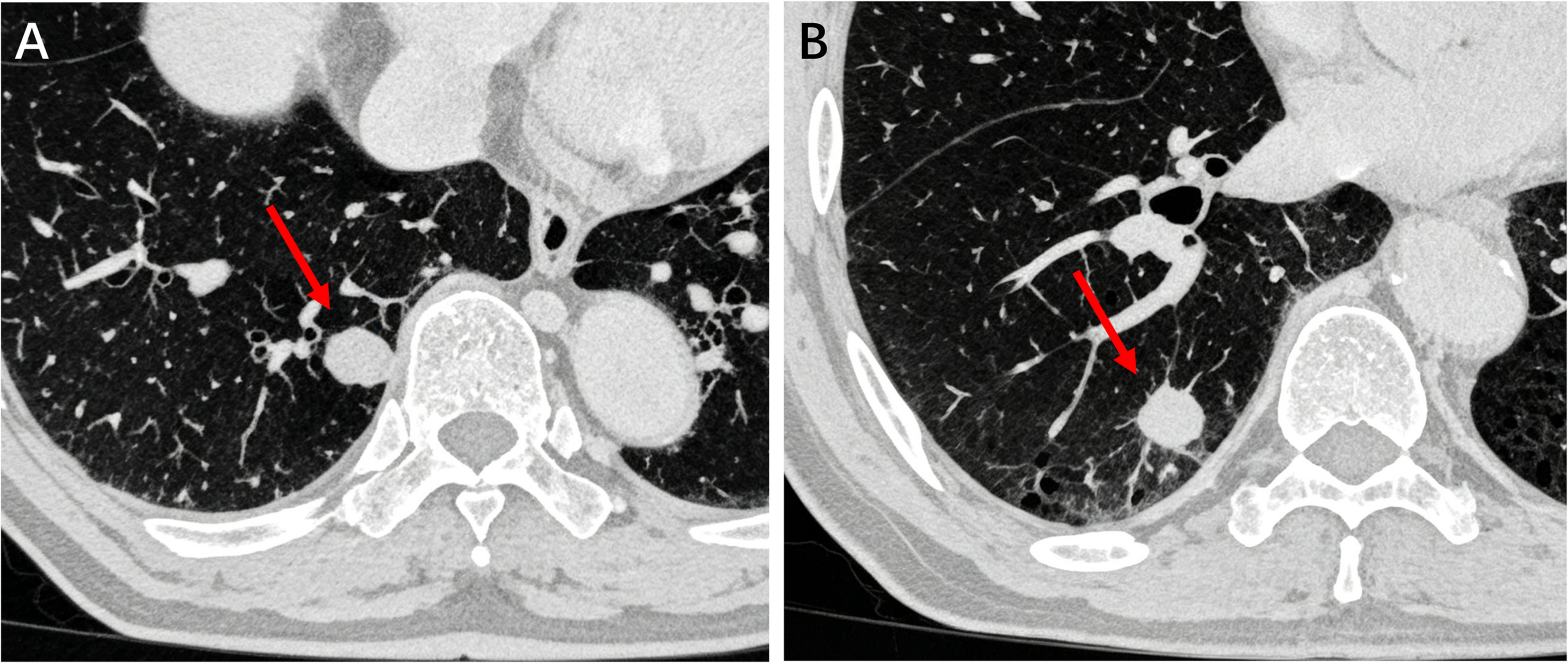
Venous-phase 70-keV VMIs, quantitative DECT parameters, and radiomics prediction probabilities of solitary pulmonary nodules (SPNs).**(A)** Benign SPN (pulmonary hamartoma, 1.65 cm, arrow) in a 63-year-old male patient. DECT metrics: V_IC_ = 2.13 mg/mL, V_NIC_ = 0.57, λHU = 0.88, V_ECV_ = 23.62%; model prediction: *P*_benign_ = 70.71%.**(B)** Malignant SPN (poorly differentiated invasive adenocarcinoma, 1.8 cm, arrow) in a 60-year-old male patient. DECT metrics: V_IC_ = 1.17 mg/mL, V_NIC_ = 0.40, λHU = 1.46, V_ECV_ = 19.10%; model prediction: *P*_malignant_ = 87.98%.

To visually manifest the clinical applicability of the proposed DECT parameters and radiomics fusion workflow, two representative cases are explicitly compared on venous-phase 70-keV VMIs (**Figure 6**). A 63-year-old male with a benign pulmonary hamartoma exhibited a high V_ECV_ of 23.62% and was correctly flagged by the model with a P_benign_ of 70.71% (**Figure 6A**). Conversely, a 60-year-old male with a poorly differentiated invasive lung adenocarcinoma presented a restricted V_ECV_ of 19.10% and was identified with a high malignancy probability (P_malignant_ = 87.98%; **Figure 6B**).

## 4 Discussion

In this study, we developed a combined model for distinguishing benign from malignant solitary pulmonary nodules by integrating a predictive model based on DECT quantitative parameters (mean diameter, V_ECV_, and V_λHU_) with a radiomics feature model. The results demonstrated that the combined model achieved excellent diagnostic performance in the test set (AUC = 0.906), significantly outperforming the standalone radiomics model. These findings suggest that the combined approach holds substantial potential for supporting clinical decision-making.

It is worth noting that V_ECV_ and V_λHU_ were identified as independent predictive factors in DECT quantitative parameters in differentiating pulmonary nodules. ECV serves as a quantitative indicator of the proportion of extracellular space in tissues, exhibiting an inverse correlation with tumor cell density (10). Pathophysiologically, benign nodules (e.g., inflammatory pseudotumors) often present with a loose tissue architecture rich in extracellular matrix, edema, or inflammatory cell infiltration, which reduces cell density and expands the extracellular compartment, thereby yielding higher ECV values. Conversely, malignant nodules are characterized by rapid cellular proliferation and dense cellular packing, which significantly constricts the interstitial space and results in a decreased ECV. Consistently, our study demonstrated that the benign group exhibited a significantly higher venous-phase ECV than the malignant group (20.02% vs. 14.72%, P< 0.001), further validating this underlying histopathological rationale.Well-differentiated lung adenocarcinomas have distinct pathological characteristics, including organized glandular structures with a regular cell arrangement and a lower cell density (11),While poorly differentiated adenocarcinomas exhibit loss of glandular architecture, characterized by disorganized and dense cellular patterns (12). λHU had intermediate to high performances to differentiate malignant nodules from benign ones, and this result is consistent with previous findings (13, 14).The results showed that venous-phase DECT parameters performed relatively well in distinguishing benign from malignant pulmonary nodules. This may be because the arterial phase primarily reflects the richness of arterial blood supply to the nodules; although many malignancies are hypervascular, certain benign lesions (such as inflammatory pseudotumors or active infections) can also exhibit significant enhancement due to acute inflammatory responses, resulting in overlapping imaging features and reduced specificity in the arterial phase. Conversely, during the venous phase, the contrast agent has more time to extravasate from the intravascular space into the interstitial tissue, better capturing the perfusion characteristics of the nodules (15, 16). Ren and Yang (17) further explored combining V_IC_ with serum biomarkers (such as vascular endothelial growth factor and carcinoembryonic antigen) to improve diagnostic accuracy. Additionally, a radiomics model based on venous-phase 70-keV monoenergetic images (V_70keV_) demonstrated strong performance in both the training (n = 146) and test (n = 63) sets.

Second, radiomics models constructed using 70-keV monoenergetic images demonstrated potential advantages. The radiomics model based on 70-keV monoenergetic images (AUC = 0.794) showed superior performance compared with the mixed-energy image model (AUC = 0.631) (*P* = 0.023). This may be related to the inherent characteristics of virtual monoenergetic images: monoenergetic images, particularly at low energy levels, can significantly enhance iodine contrast, making blood vessels and iodine-rich tissues (such as tumors) more conspicuous. This enhancement facilitates better visualization of small lesions or subtle abnormalities (18–20), resulting in clearer image details (20, 21). In radiomics analysis, the optimal energy level varies across studies owing to differences in clinical tasks, equipment, and reconstruction algorithms. For example, Xu et al. (22) used radiomic features from 65-keV images to evaluate pulmonary nodules and found significant diagnostic value, whereas Chen et al. (23) demonstrated that radiomics models based on 40- and 100-keV monoenergetic images achieved excellent performance in distinguishing lung adenocarcinoma from squamous cell carcinoma.

Further studies are warranted to explore large-scale, multi-center cohorts to address potential variations associated with institution-specific DECT protocols and vendor technologies. Notably, while the high training AUC (0.998) reflects the robust fitting capacity of the machine-learning algorithms when exposed to high-dimensional radiomics features, the clinical-DECT combined model maintained an excellent and stable diagnostic performance on the independent test dataset, yielding an AUC of 0.906. This confirms that the implementation of LASSO regularization and internal 5-fold cross-validation during the training phase successfully minimized the risk of catastrophic overfitting, ensuring robust generalizability for noninvasive SPN characterization.

## 5 Limitations

Several limitations of this study should be acknowledged. First, this study is a single-center retrospective investigation. Although internal validation was performed, the generalizability and robustness of the predictive models require further verification using independent external cohorts from multiple centers.

Second, the malignant group was composed exclusively of adenocarcinomas and squamous cell carcinomas, with no other rare malignant subtypes included. Additionally, a notable class imbalance existed between the malignant (n = 166) and benign (n = 43) groups. Although this distribution reflects the real-world clinical prevalence of surgical candidates in a tertiary hospital setting, future studies with larger and more balanced cohorts are needed to minimize potential classifier bias.

Third, this study only included surgically resected and pathologically confirmed pulmonary nodules, thus excluding nodules<10 mm,patients managed conservatively, and calcified nodules.

Fourth, a dimensional inconsistency exists within our methodological pipeline, wherein 2D ROI slice-averaging was used for conventional DECT quantitative parameter measurements while 3D VOI full-lesion segmentation was employed for radiomics feature extraction. Although this dual-approach design satisfies the respective localized and high-dimensional technical requirements of each modality, it may theoretically introduce cross-dimensional selection bias. Future multi-center prospective investigations should evaluate the feasibility of standardized 3D volume-based metrics across both conventional spectral CT and advanced machine-learning workflows to entirely eliminate cross-dimensional variation.

Consequently, the findings may not be generalizable to low-dose CT screening populations or incidentally detected sub-centimeter nodules. Dedicated studies in screening cohorts are required to validate the diagnostic value of DECT parameters in these specific populations.

## 6 Conclusion

Overall, the findings demonstrate that the DECT parameter–radiomics combined model can effectively differentiate between benign and malignant solitary pulmonary nodules, indicating promising potential as a noninvasive tool to refine localized clinical decision-making. Extensive external validation and prospective studies remain essential before clinical deployment.

## Data Availability

The original contributions presented in the study are included in the article/**Supplementary Material**. Further inquiries can be directed to the corresponding author.
