# Supplementary material for: Evaluation of a dual-energy computed tomography parameter –radiomics combined model for solitary pulmonary nodules
Source: Front Oncol. 2026 Jul 22;16:1778055. doi: 10.3389/fonc.2026.1778055 (PMC13437286; doi:10.3389/fonc.2026.1778055)
Supplement: Supplementary file 1 [file Table1.docx]

**Supplementary Table 1. Interobserver Agreement Analysis of DECT Quantitative Parameters**

| Parameter | Phase | ICC | 95% CI | Agreement Level* |
| --- | --- | --- | --- | --- |
| Iodine concentration (IC) | Arterial | 0.81 | 0.73–0.88 | Good |
| Normalized iodine concentration (NIC) | Arterial | 0.83 | 0.75–0.90 | Excellent |
| Normalized effective atomic number (nZeff) | Arterial | 0.78 | 0.70–0.86 | Good |
| Spectral HU slope (λHU) | Arterial | 0.76 | 0.69–0.84 | Good |
| Extracellular volume fraction (ECV) | Arterial | 0.84 | 0.77–0.91 | Excellent |
| Iodine concentration (IC) | Venous | 0.86 | 0.79–0.92 | Excellent |
| Normalized iodine concentration (NIC) | Venous | 0.88 | 0.81–0.92 | Excellent |
| Normalized effective atomic number (nZeff) | Venous | 0.80 | 0.72–0.88 | Good |
| Spectral HU slope (λHU) | Venous | 0.77 | 0.70–0.85 | Good |
| Extracellular volume fraction (ECV) | Venous | 0.85 | 0.78–0.91 | Excellent |

*Classification of Agreement Levels: ICC < 0.50 = Poor; 0.50–0.75 = Moderate; 0.75–0.90 = Good; > 0.90 = Excellent.
